# Characterization and expression of a long neuropeptide F (NPF) receptor in the Chagas disease vector *Rhodnius prolixus*

**DOI:** 10.1371/journal.pone.0202425

**Published:** 2018-08-16

**Authors:** Laura Sedra, Jean-Paul Paluzzi, Angela B. Lange

**Affiliations:** 1 Department of Biology, University of Toronto Mississauga, Mississauga, ON, Canada; 2 Department of Biology, York University, Toronto, ON, Canada; Biocenter, Universität Würzburg, GERMANY

## Abstract

In this study, a long neuropeptide F receptor of the blood-feeding hemipteran, *Rhodnius prolixus* (RhoprNPFR) has been cloned and characterized. Approximately 70% of the RhoprNPFR deduced protein sequence is identical to that of other hemipteran NPFRs. RhoprNPFR has seven highly-conserved transmembrane domains, two cysteine residues in the 2^nd^ and 3^rd^ extracellular loops that likely form a disulfide bond integral for maintaining the structure of the receptor, and a conserved DRY motif after the third transmembrane domain. All of these characteristics are typical of class A rhodopsin-like GPCRs. The receptor transcript is predominantly expressed in the central nervous system (CNS) and gut of both fifth instar and adult *R*. *prolixus*. Using fluorescent *in situ* hybridization (FISH), we identified six bilaterally-paired large median neurosecretory cells (approximately 30μm in diameter) in the brain that express the RhoprNPFR mRNA transcript. We also found RhoprNPFR transcript expression in endocrine cells in the anterior midgut of fifth instars, as well as in putative pre-follicular cells present in the germarium and between developing oocytes, and in the nutritive cord. These results suggest that RhoprNPFR may play a role within the CNS, and in digestion and possibly egg production and/or egg development in *R*. *prolixus*.

## Introduction

Neuropeptides and their receptors regulate many physiological processes such as digestion, metabolism, development and reproduction. Many of these functions are modulated by G protein-coupled receptors (GPCRs), the largest and most diverse group of membrane receptors [1]. Evolutionary conservation has been deduced for many ligand-receptor pairs, including the vertebrate neuropeptide Y (NPY) [2]. The orthologous neuropeptide and receptor among invertebrates is neuropeptide F (NPF) [2, 3]. Recently, the NPF cDNA was cloned in the blood-gorging kissing bug, *Rhodnius prolixus*, and the neuropeptide was determined to be 42 amino acids in length with a truncated peptide composed of the last 8 amino acids (truncated RhoprNPF) found to be biologically active [4]. This active C-terminal region was also observed in *Schistocerca gregaria* and found to regulate food intake and body weight [5]. Expression of the RhoprNPF mRNA transcript was found in medial neurosecretory cells of the central nervous system (CNS), as well as in cells along the longitudinal muscle fibers of the lateral oviduct [4]. Although RhoprNPF did not have an effect on oviduct muscle contraction, we recently showed that this neuropeptide is responsible for regulating egg production in *R*. *prolixus* [4].

The first NPFR (DmNPFR 1) was cloned in *Drosophila melanogaster* [6] and found in neurons in the CNS of late third instar larva as well as in endocrine cells in the midgut using *in situ* hybridization techniques [6]. An NPFR was also characterized in *Anopheles gambiae* and the Ang-NPFR transcript was shown to be present in the head, thorax and abdomen of all life stages [7]. In *Aedes aegypti*, an analysis investigating receptors structurally related to vertebrate neuropeptide Y-like receptors provided evidence that at least two receptors are functionally responsive to NPF [8], although their expression profiles and function in the mosquito were not determined. Recently, an NPF and NPY receptor was identified in the locust, *Locusta migratoria*, which were shown to be essential for locomotor activity changes related to phase transition [9]. Beyond insect species, NPFR was cloned in the pond snail, *Lymnaea stagnalis*; however, it was first described as an NPY receptor [10]. Other than these studies, very little is known about the NPF receptor in molluscs or arthropods.

In this study, we molecularly identified an NPFR cDNA sequence in *R*. *prolixus* and determined its spatial expression profile at the transcript level in fifth instar and adult insects. Quantitative RT-PCR (qPCR) was also used to determine the expression profile of the NPFR transcript in the female and male reproductive tracts. Finally, NPFR transcript expression was localized to neurons of the CNS, putative follicular cells in the ovaries and within the nutritive cord, as well as, endocrine cells in the midgut and cells in the hindgut using fluorescent *in situ* hybridization (FISH).

## Materials and methods

### Animals

Fifth instar and adult *R*. *prolixus* were obtained from a colony fed on defibrinated rabbit blood (Hemostat Laboratories, Dixon, CA, USA; supplied by Cedarlane, Burlington, ON, Canada). A blood meal is required for molting and sexual maturation. The colony was maintained in an incubator at 25°C and 60% humidity. Experimental insects were kept in an incubator with a 12h:12h light/dark cycle at 28°C and 50% humidity. All insects (both fifth instars and adults) used for the expression profile as well as the *in situ* experiments were 4 weeks old after molting, unfed, and not mated.

### Chemicals

All gene-specific primers (GSP) and probes used were designed with Geneious Bioinformatics Software v.4.7.6 (Biomatters, Aukland, New Zealand) and primers were synthesized by Sigma-Aldrich (Oakville, Ontario, Canada) and made up in RNase-free double-distilled water and stored at -20°C.

### Cloning of the RhoprNPFR cDNA sequence

The previously characterized amino acid sequence of the long neuropeptide F receptor from the mosquito *A*. *gambiae* (Ang-NPFR: AY579078) [7] was used to screen the *R*. *prolixus* genome via a BLAST search (Basic Local Alignment Search Tool). All *in silico* work was completed in Geneious Bioinformatics Software, and a putative NPFR sequence was defined (Supercontig: GL563029) in the *R*. *prolixus* genome [11, 12]. Gene-specific primers were designed to clone the open reading frame (ORF) (Table 1A). Predicted splice sites were taken into consideration when designing any forward or reverse primers (http://www.fruitfly.org/seq_tools/splice.html) [13]. Amplification and cloning conditions were similar to previously described methods in [4]. Size and purity of all amplified products were checked on a 1.2% agarose gel stained with RedSafe^TM^ nucleic acid staining solution (iNtRON Biotechnology, New Jersey, USA) and quantified using a Nanodrop UV spectrophotometer (Thermo Scientific, Wilmington, Delaware, USA). Samples were then sent for sequencing at the Hospital for Sick Children (The Centre for Applied Genomics, Sick Kids, Toronto, Ontario, Canada). Sequencing results were then further analyzed using Geneious Bioinformatics Software to confirm base accuracy.

**Table 1:** Gene specific primers (GSPs) designed to clone the RhoprNPF receptor.

**A) ORF primers (5' to 3')**	
NPFR-FOR1	CAAAAACGACGATCACAATGTTG
NPFR-REV1	CTGTATAAGTAGTGGCCGGTTGTTG
**B) 5' and 3' RACE primers**	
raceNPFR-REV1	TCACAATGTATAAATTCCGAGCAG
raceNPFR-REV2	TACCGACAACAATTAATAAAGCGTACAG
raceNPFR-REV3	CAACATTGTGATCGTCGTTTTTG
raceNPFR-FOR1	CAACAACAGACCACAAATGCAC
raceNPFR-FOR2	CAAGTCCAACGATAATGTTATGCC
raceNPFR-FOR3	CAACAACCGGCCACTACTTATACAG
**C) Complete Receptor**	
fullNPFR-FOR1	GACGAAACTGCCCCATAAC
fullNPFR-REV1	GTAGATTTACAAAATGTCACATTTAGTTTTATAC

Modified 5' and 3' rapid amplification of cDNA ends (RACE) was used to clone the ends of the ORF as well as a portion of the 5’ and 3’ untranslated region (UTR) (Table 1B). An *R*. *prolixus* fifth instar CNS cDNA library [14] was used as a template, and 5' RACE gene-specific reverse primers as well as library plasmid forward primers were used to extend the 5' end of the sequence. This was similarly carried out for the 3’ end. These products were then used as the cDNA template for further nested PCRs as previously described in [4].

To clone the full receptor coding sequence, GSPs were designed at the start and end of the complete ORF, and products were sent for sequencing to confirm that the full receptor had been cloned correctly (Table 1C). An iProof^TM^ High Fidelity DNA polymerase (BioRad, Ontario, Canada) was used to amplify the full sequence and confirm base accuracy of the complete and correct RhoprNPFR.

### Sequence analysis of the RhoprNPF receptor

The complete cloned nucleotide sequence was queried in a BLAST search against the *R*. *prolixus* genome using the Geneious software. All contigs were then aligned, and the size of the introns were determined. Splice-sites at intron-exon boundaries were confirmed by comparing NNSPLICE 0.9 predictions (http://www.fruitfly.org/seq_tools/splice.html) [13] and the cloned cDNA sequence. N-linked glycosylation sites on the N-terminal extracellular chain were predicted using the NetNGlyc 1.0 server (http://www.cbs.dtu.dk/services/NetNGlyc/) [15]. The TMHMM server v. 2.0 (TransMembrane Hidden Markov Model) was used to determine the hydrophobic transmembrane helical domains within the receptor (http://www.cbs.dtu.dk/services/TMHMM/) [16]. The DiANNA1.1 web server was used to predict potential cysteine residues within the ORF that can form a disulfide bond (http://clavius.bc.edu/~clotelab/DiANNA/). In order to predict potential phosphorylation sites on the intracellular loops and C-terminal cytoplasmic chain, the NetPhos 2.0 server was utilized (http://www.cbs.dtu.dk/services/NetPhos/) [17]. Lastly, to gain a fuller understanding of the correct folding and final tertiary structure of this protein, SWISS-MODEL software was used to match the conserved portions of the cloned RhoprNPFR against previously characterized receptor sequences with known tertiary structures (http://swissmodel.expasy.org) [18].

### Multiple sequence alignment and phylogenetic analysis of NPFR

Multiple arthropod and vertebrate cloned and predicted sequences of NPFR and NPYR were aligned against the deduced amino acid sequence of RhoprNPFR using ClustalW server (sequences defined in the figure captions; http://www.ch.embnet.org/software/ClustalW.html). The alignment was then imported onto the BOXSHADE 3.21 server (http://www.ch.embnet.org/software/BOX_form.html), and conserved sequences were shaded so that identical amino acid residues were highlighted in black and amino acid positions with similar chemical characteristics in gray following the 60% majority rule. Residues that were 100% conserved across all 14 species were manually determined and denoted by an asterisk.

Various insect NPF receptors previously identified or predicted as well as closely related receptor subtypes belonging to the short NPF and NPY receptor subfamilies were imported into and analyzed using MEGA 6.06 (Molecular Evolutionary Genetics Analysis) [19]. Evolutionary relationships amongst the various subtypes of insect NPF-related receptors was determined through phylogenetic analyses using the maximum likelihood method based on the Jones-Thornton-Taylor matrix-based model [20] and neighbour-joining method [21]. In order to determine confidence statistics for the observed phylogeny, bootstrap analysis was carried out using 1000 replicates [22].

### Total RNA extraction from various tissues and cDNA synthesis

The CNS comprised of brain, suboesophageal and prothoracic ganglia, and the mesothoracic ganglionic mass was isolated by microdissection along with various peripheral tissues/organs (e.g. salivary gland, dorsal vessel, foregut, anterior midgut, posterior midgut, hindgut, Malpighian tubules, fat body, ovaries and testes) of fifth instar and adult *R*. *prolixus* and stored in RNA later solution (Ambion, Carlsbad, California, USA). Similar dissections were completed for adult reproductive tissues of females (eg. ovaries, oviduct/spermathecae and bursa/cement gland) and males (eg. testes, vas deferens/accessory glands and seminal vesicle/ejaculatory duct). There were three biological replicates for every dissected tissue sample each containing pooled samples from at least 10 insects. Tissues were then processed as previously described in [4] using the PureLink® RNA mini-kit (Ambion, Carlsbad, California, USA), which included DNase treatment to remove any potential contaminating genomic DNA. The purity and concentration of each RNA sample was determined by the Nanodrop. Total RNA isolated from these dissections was used for the spatial profiling of the RhoprNPFR transcript. For each tissue/organ sample, 200ng of RNA was aliquoted to synthesize cDNA using the iScript^TM^ Reverse Transcription Supermix for RT-qPCR (Bio-Rad Laboratories Ltd., Mississauga, Ontario, Canada). Following synthesis, cDNA was diluted 10-fold and used as template for quantitative PCR (qPCR).

### Expression profile of RhoprNPFR transcript across various tissues

All spatial profiling experiments were performed on an MX3005P Quantitative PCR system (Stratagene, La Jolla, California, USA) as described earlier in [4]. GSPs were designed over exon/exon boundaries to determine the RhoprNPFR transcript abundance in each sample (Table 2A). Three reference genes were used for analysis (ribosomal protein 49, β-actin and α-tubulin) as previously described in [4]. Efficiencies for all primers used were determined (Table 2B), and the ΔΔC_t_ method of analysis was used to determine the fold-differences of RhoprNPFR transcript abundance in the various tissues relative to testis in fifth instars and adults [23].

**Table 2:** Primers used to determine the expression profile of RhoprNPFR in fifth instar and adult tissues using qPCR. In addition, primers used to synthesize sense (control) and antisense (experimental) DIG-labelled RNA probes to detect RhoprNPFR mRNA via FISH.

A) GSPs for qPCR*		
**Name**	**Sequence**	**Primer Efficiency**
qPCR-NPFR-FOR1	CAGTCGTCTTCTTCCAGATAGTGG	90%
qPCR-NPFR-REV2	CGAACAGTACTGCTACCGCTG	
B) Reference gene primers for qPCR		
rp49-qPCR-F	GTGAAACTCAGGAGAAATTGG	100%
rp49-qPCR-R	AGGACACACCATGCGCTATC	
Actin5C-qPCR-F	AGAGAAAAGATGACGCAGATA	98.5%
Actin5C-qPCR-R	ATATCCCTAACAATTTCACGTT	
alphaTUB-qPCR-F	GTGTTTGTTGATTTGGAACCTA	97.6%
alphaTUB-qPCR-R	CCGTAATCAACAGACAATCTTT	
**C) GSPs for sense strand**		
T7-NPFR-FOR1	TAATACGACTTATAGGGAGACAAAAACGACGATCACAATGTTG	
iNPFR-REV1	GGATATGACTCGGCGTC	
**D) GSPs for antisense strand**		
NPFR-FOR1	CAAAAACGACGATCACAATGTTG	
T7-iNPFR-REV1	TAATACGACTTATAGGGAGAGGATATGACTCGGCGTC	

* GSPs designed on exon-exon boundaries to avoid amplification of genomic DNA.

With a particular interest in the adult reproductive tissues, transcript expression of RhoprNPFR was determined throughout the adult male and female reproductive system and plotted relative to transcript abundance in ovaries. All spatial profiling samples were run using SsoFast^TM^ EvaGreen® Supermix with Low ROX (BIO-Rad Laboratories Ltd., Mississauga, Ontario, Canada). Transcript abundance was normalized to the three chosen reference genes for all tissues sampled. Two technical replicates were performed (as well as a no template control) for each biological replicate.

### Fluorescent *in situ* hybridization (FISH)

Sense (T7-NPFR-FOR1 and iNPFR-REV1) and antisense (NPFR-FOR1 and T7-iNPFR-REV1) gene-specific primers were designed within the RhoprNPFR open reading frame to prepare the cDNA template for the synthesis of the RNA probes used for fluorescent *in situ* hybridization (Table 2C and 2D). A DIG/RNA labelling kit (Roche Applied Science, Mannheim, Germany) was used to transcribe the DIG-labelled sense and antisense probes. Following the *in vitro* RNA probe synthesis reaction, DNase I was used to remove any remaining template DNA, and RNA probe quality was verified on a 1.2% agarose gel using electrophoresis and quantified by Nanodrop UV spectrometer. DIG-labelled RNA probes were then aliquoted and stored at -20°C.

Tissues from fifth instar and adult *R*. *prolixus* (CNS, gut and adult female reproductive tract) were dissected and incubated for 1h in 2% paraformaldehyde made up in PBST (1xPBS and 0.1% Tween-20: BioShop® Canada Inc., Burlington, ON, Canada) at room temperature. Tissues were washed with PBST and then endogenous peroxidase activity was quenched with 1% H_2_O_2_ and tissues permeabilized with 4% Triton-X prepared in PBST. Samples were processed as previously described by [4] using the TSA amplification kit (Molecular Probes, Life Technologies, MA, USA), with the only modification being the RhoprNPFR-specific DIG-labelled RNA probes in which the tissues were incubated. For observation, tissues were mounted in 100% glycerol on glass slides (Sigma Aldrich, Oakville, ON, Canada) and images were acquired on a Zeiss LSM 510 Confocal Laser Microscope (Carl Zeiss, Jena, Germany). Separate preparations were simultaneously incubated with sense probes for every experiment as a negative control.

## Results

### NPF receptor in *R*. *prolixus*

**Fig 1:**
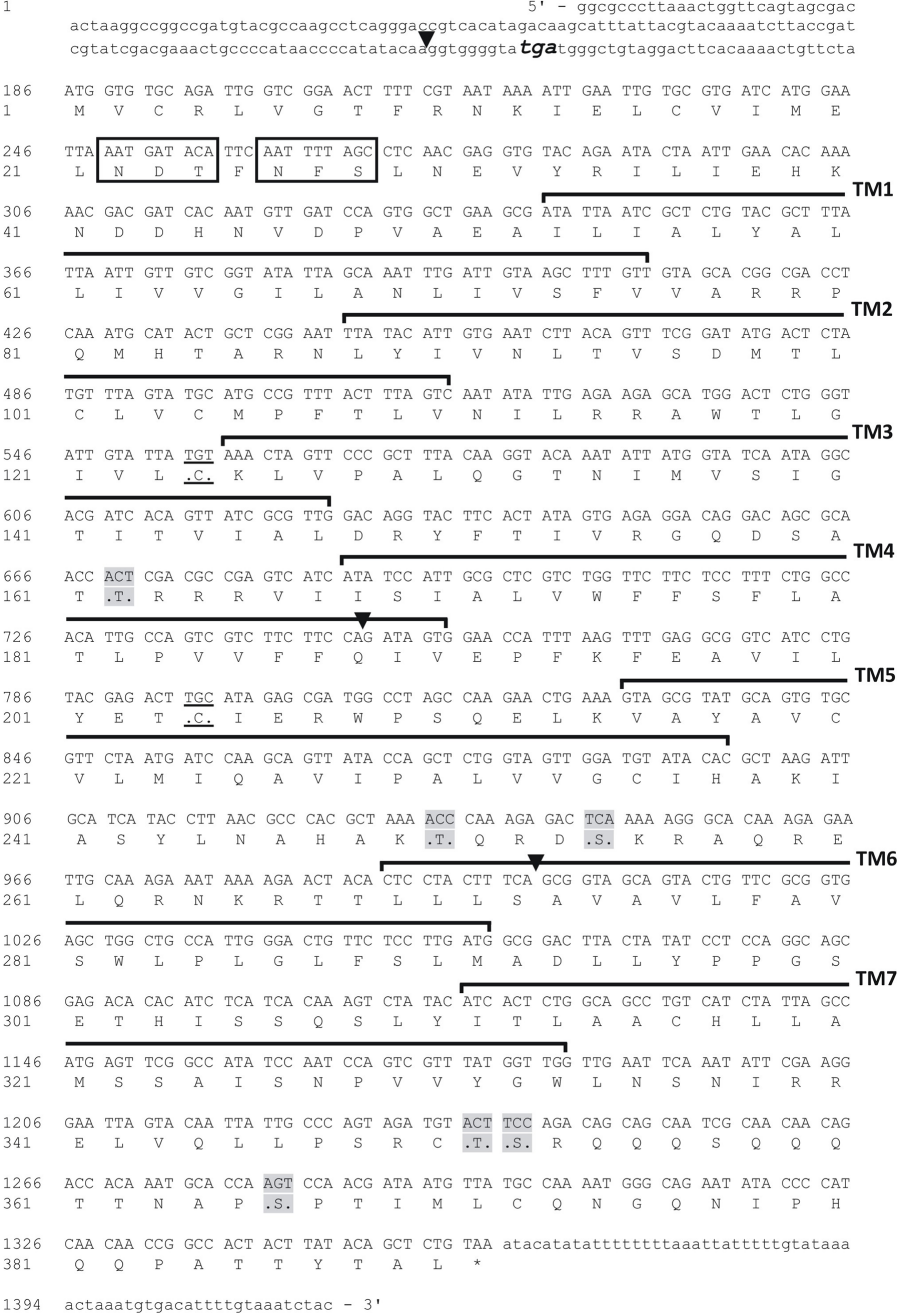
cDNA sequence and the deduced amino acid sequence of NPFR in *R*. *prolixus*. Nucleotide and amino acid sequence of the open reading frame (ORF), where numbers for each sequence are provided on the left. The ORF starts with the ATG start codon and the asterisk denotes the stop codon (TAA). The full ORF is 1173bp long and yields a deduced receptor protein comprised of 390 amino acid residues (GenBank accession #: KM882822). The stop codon before the methionine start codon is bolded, enlarged in font and italicized in the 5’ untranslated region (UTR). Exon-exon boundaries are represented by the downward solid arrowheads. The seven hydrophobic transmembrane domains are outlined and labeled (TM1-7) and both cysteine residues predicted to be involved in a disulfide bond are underlined. Potential *N*-linked glycosylation sites are boxed at the amino-terminal chain, whereas the predicted phosphorylation sites on the cytoplasmic loops are shaded in gray.

**Fig 2:**
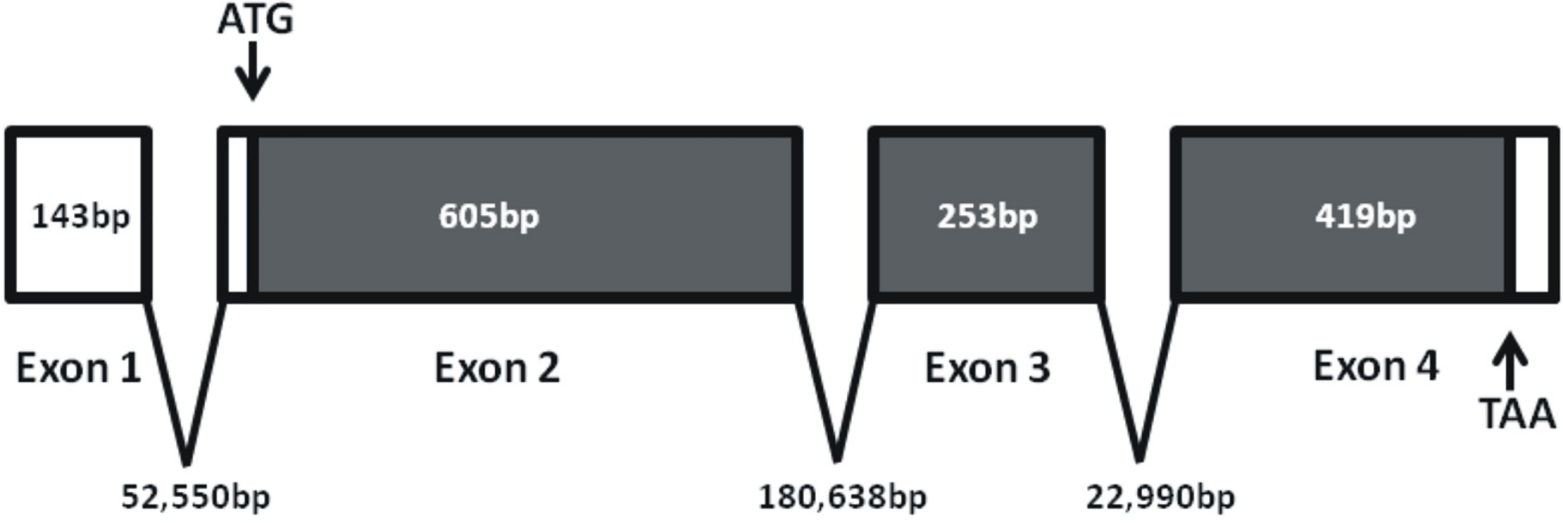
Exon-intron map of the *RhoprNPF* receptor. A graphical representation of the gene comprised of boxed exons, where every break represents a splice site. White boxes denote the 5' and 3' UTRs and the gray boxes represent the ORF, based on splice site predictions and BLAST analysis against the *Rhodnius prolixus* genome. The start and stop codons are labeled with arrows. The ORF contains a total of 3 exons (drawn to scale). Numbers denote exon and intron sizes in nucleotide base pairs, where intron sizes are not drawn to scale.

**Fig 3:**
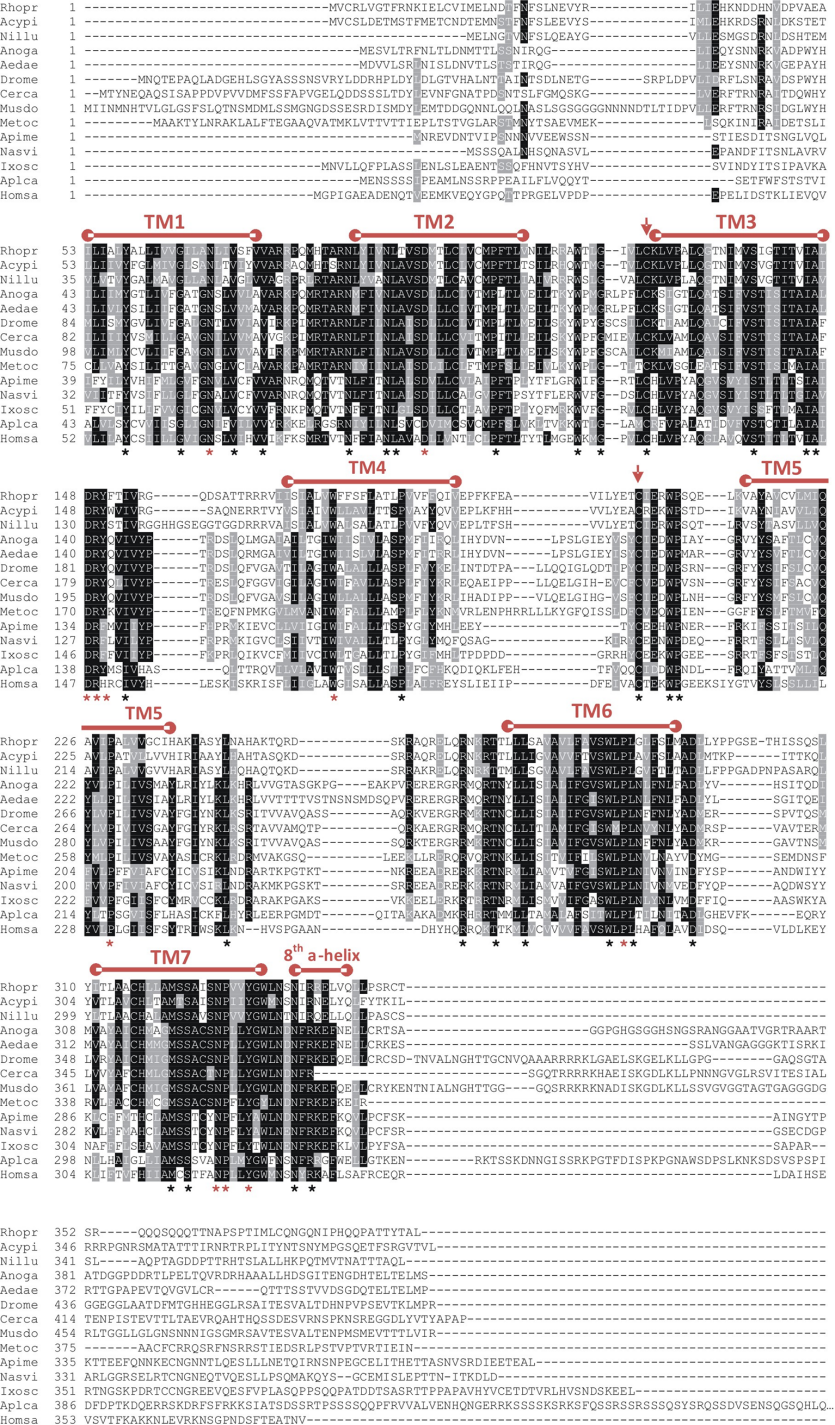
Amino acid sequence alignment of NPFR identified or predicted in 13 other species, using ClustalW. The predicted seven transmembrane regions (TM 1–7) are indicated above the alignment. The two conserved cysteine residues used in a disulfide bond are denoted by downward pointing arrows. Residues that are 100% conserved across all 14 species are denoted with a black asterisk, whereas residues that are classic identifiers of GPCRs and are commonly conserved were represented by a red asterisk. Following the 60% majority rule, identical amino acids are shaded in black and similar amino acids are shaded in gray. NPF/NPY-like receptor sequences from *R*. *prolixus* (Rhopr), *A*. *pisum* (Acypi: XM_001943673), *N*. *lugens* (Nillu: BAO01088.1), *A*. *gambiae* (Anoga: AY579078), *A*. *aegypti* (Aedae: KC439539), *D*. *melanogaster* (Drome: AF36440), *Ceratitis capitata* (Cerca: XM_004534122), *Musca domestica* (Musdo: XM_005182221), *Metaseiulus occidentalis* (Metoc: XM_003739518), *Apis mellifera*, (Apime: XM_001123033), *Nasonia vitripennis* (Nasvi: XM_001601922), *I*. *scapularis* (Ixosc: KC439541), *A*. *californica* (Aplca: XM_005089570) and *Homo sapiens* (Homsa: AY2365401) were used.

The long neuropeptide F receptor of *R*. *prolixus* has been cloned, and the coding sequence is composed of 1173bp yielding a deduced receptor sequence comprised of a 390 amino acid polypeptide (Fig 1; GenBank Accession #—KM882822). Using 5' and 3' RACE we were able to elucidate 72 nucleotides of the 5' untranslated region (UTR) and 62 nucleotides of the 3' UTR (Fig 1). The RhoprNPFR ORF is composed of three exons (605bp, 253bp and 419bp) and two introns (180,638bp and 22,990bp) (Fig 2). RhoprNPFR appears to be a classic rhodopsin-like GPCR, with seven hydrophobic transmembrane domains, three extracellular loops and three cytoplasmic loops (Fig 1). RhoprNPFR shares various conserved amino acid motifs in the transmembrane regions with other rhodopsin GPCRS (denoted by red asterisks; Fig 3) [24]. The cytoplasmic end of the third transmembrane domain contains the highly-conserved DRY motif that is typical of rhodopsin GPCRs [25]. Lastly, *R*. *prolixus* as well as many other invertebrate and vertebrate species share an NPXXY motif in the seventh transmembrane domain that is also typical of GPCRs (Fig 3) [24]. RhoprNPFR has a short *N*-terminus that contains two predicted glycosylation sites and six predicted phosphorylation sites on the cytoplasmic loops and *C*-terminal end of the receptor (Fig 1). There are two cysteine residues at positions 124 and 204 that exhibit significant conservation across all species analyzed and are predicted to form a disulfide bond between the first two extracellular loops for structural purposes. An 8^th^ α-helical chain is predicted at the end of the receptor where not all the residues are hydrophobic and, therefore, it is not a transmembrane chain.

### Alignment and phylogenetic analysis of RhoprNPFR

The translated RhoprNPFR ORF was aligned with NPFR-related sequences from 10 other insect species, one arachnid (*Ixodes scapularis*), one mollusk (*Aplysia californica*) and a vertebrate (*Homo sapiens*). The *N*-linked glycosylation sites appear to be conserved in insects, whereas all seven transmembrane domain regions are evolutionarily conserved across the various phyla. The 8^th^ α-helix is also well conserved. Compared to other Hemipterans, such as *Acyrthosiphon pisum* and *Nilaparvata lugens*, RhoprNPFR exhibits 72% and 77% sequence similarity, respectively. RhoprNPFR was also found to be 58% and 65% identical to *A*. *pisum* and *N*. *lugens*, respectively. When comparing RhoprNPFR to representative sequences from Diptera, mollusk and vertebrate species, approximately 43% of the amino acid residues exhibit amino acid similarity and 29% are identical across all 13 species (Fig 3).

**Fig 4:**
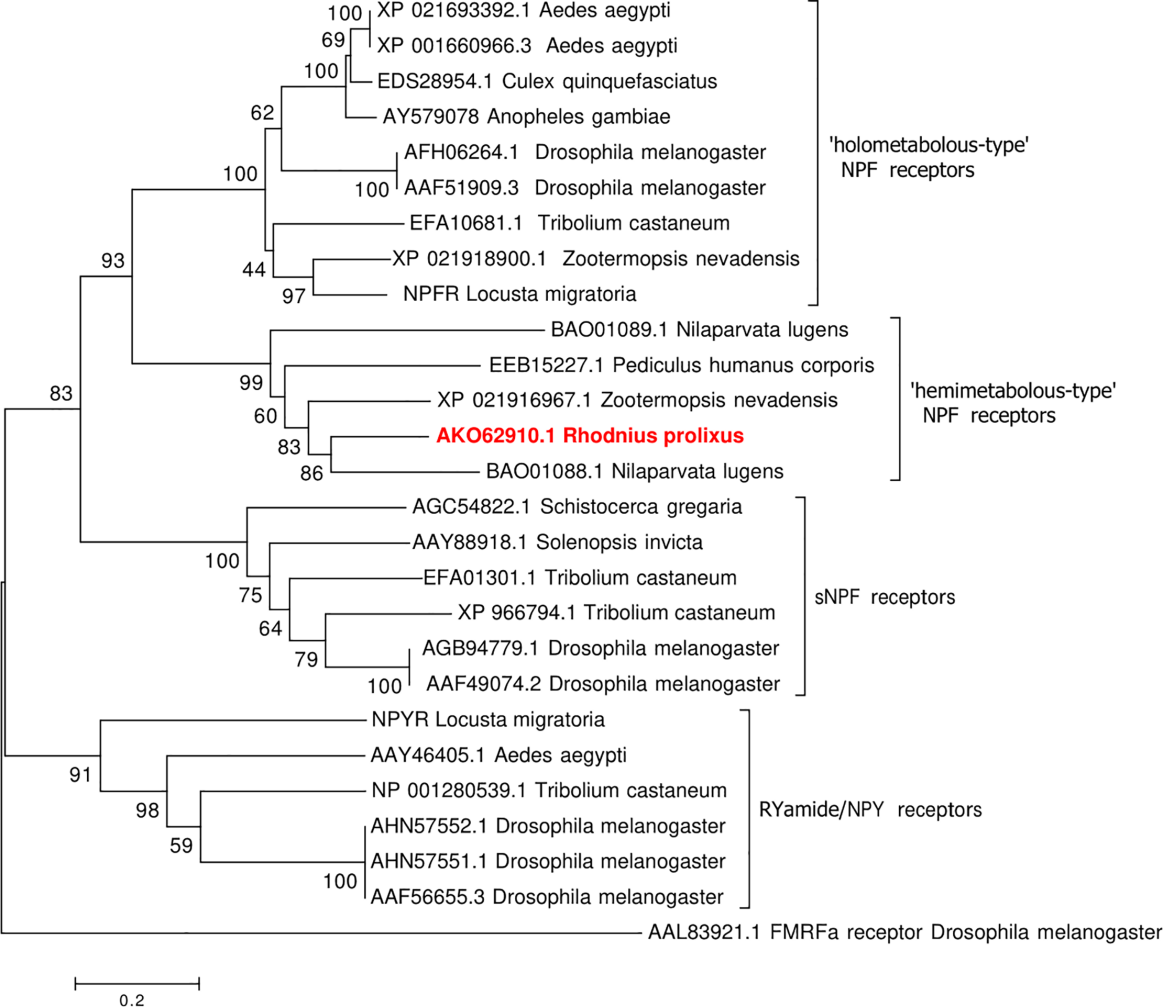
Phylogenetic analysis of RhoprNPFR with related receptor sequences from other insects, including members of the NPF and sNPF receptor families. A rooted phylogenetic tree depicting the receptor sequence relationship inferred using the neighbour-joining method. The numbers at each node represent the percentage of replicate trees in which the associated receptor sequences clustered together in the bootstrap test (1000 replicates). The tree is drawn to scale, with branch lengths representing the evolutionary distances, which are in the units of the number of amino acid substitutions per site. The final analysis involved 223 amino acid positions with all positions containing gaps and missing data being excluded from the analysis. Each receptor sequence is represented by the GenBank accession numbers and species name from which the sequence originated.

A robust phylogenetic analysis of insect-specific NPFR-related sequences using either maximum likelihood (S1 Fig) and neighbour-joining methods (Fig 4) yielded trees with similar topologies and identical clustering of the selected receptor sequences into four monophyletic groups strongly supporting the observed evolutionary relationships amongst the various NPF-related receptor sequences examined. Specifically, the *R*. *prolixus* NPFR identified herein belongs to a clade (the ‘hemimetabolous-type’) comprised of NPF receptor sequences that are distinct from a second monophyletic group (the ‘holometabolous-type’) comprising NPF receptors from several species of Diptera and other holometabolous insects including the beetle, *Tribolium castaneum*, but that also includes receptors from the hemimetabolous insects, the locust (*L*. *migratoria*) and the termite, *Zootermopsis nevadensis*. Interestingly, the termite *Z*. *nevadensis* contains an NPF receptor in both the hemimetabolous- and holometabolous-type NPF receptor clades. The hemimetabolous-type NPFR clade also contains two NPF receptors from the brown plant hopper, *N*. *lugens*, a receptor from the human body louse, *Pediculus humanus corporis*, and as mentioned earlier, a second NPF-like receptor from the termite, *Z*. *nevadensis*. A sister group to the two NPF receptor clades is comprised of receptors functionally responsive to short NPF and includes member sequences from the desert locust, *S*. *gregaria*, the beetle, *T*. *castaneum*, the red imported fire ant, *Solenopsis invicta* and the fruit fly, *D*. *melanogaster*. The *D*. *melanogaster* FMRFa receptor was set as the outgroup to root the tree.

### Expression profiling of RhoprNPFR

**Fig 5:**
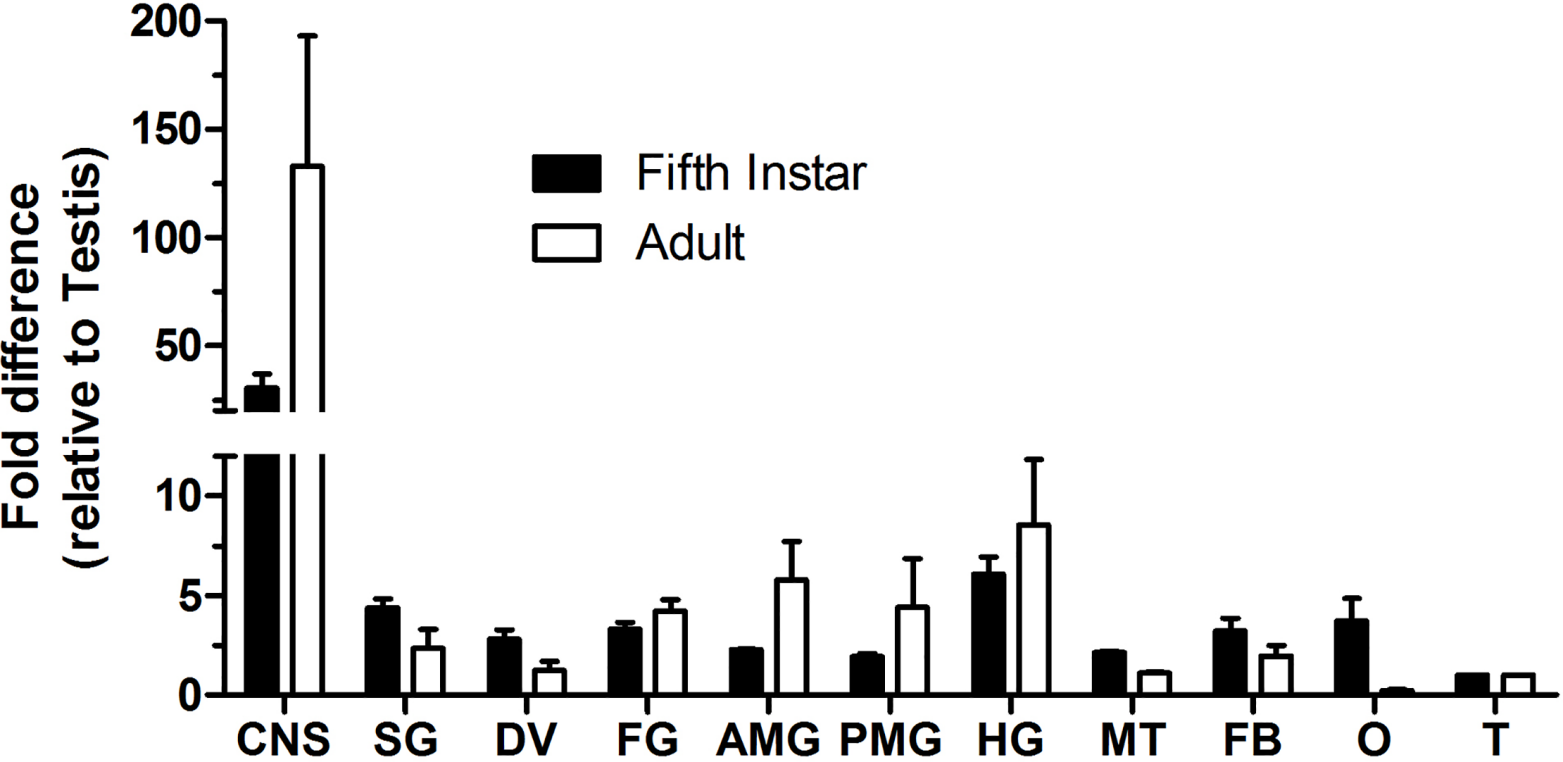
Spatial expression profile of the RhoprNPFR transcript in different organs/tissues of fifth instar and adult *R*. *prolixus*. RhoprNPFR transcript levels were measured in the CNS as well as various peripheral tissues in fifth instar (black) and adult (white) *R*. *prolixus*. The fold-difference in transcript expression observed is relative to fifth instar and adult testes. Relatively high expression of the transcript was found in the CNS as well as the digestive system (SG, FG, AMG, PMG and HG) of fifth instars and adults. Data points are mean ± standard error of the mean (SEM) of 3 biological replicates. Abbreviations: CNS, central nervous system; SG, salivary gland; DV, dorsal vessel; FG, foregut; AMG, anterior midgut; PMG, posterior midgut; HG, hindgut; MT, Malpighian tubules; FB, fat bodies; O, ovaries; T, testes.

The spatial expression of the RhoprNPF receptor transcript was observed across various tissues in two developmental stages: fifth instars and adults. In both stages, receptor mRNA was predominantly enriched in the CNS as well as digestive system (salivary gland, foregut, anterior midgut, posterior midgut and hindgut) (Fig 5).

**Fig 6:**
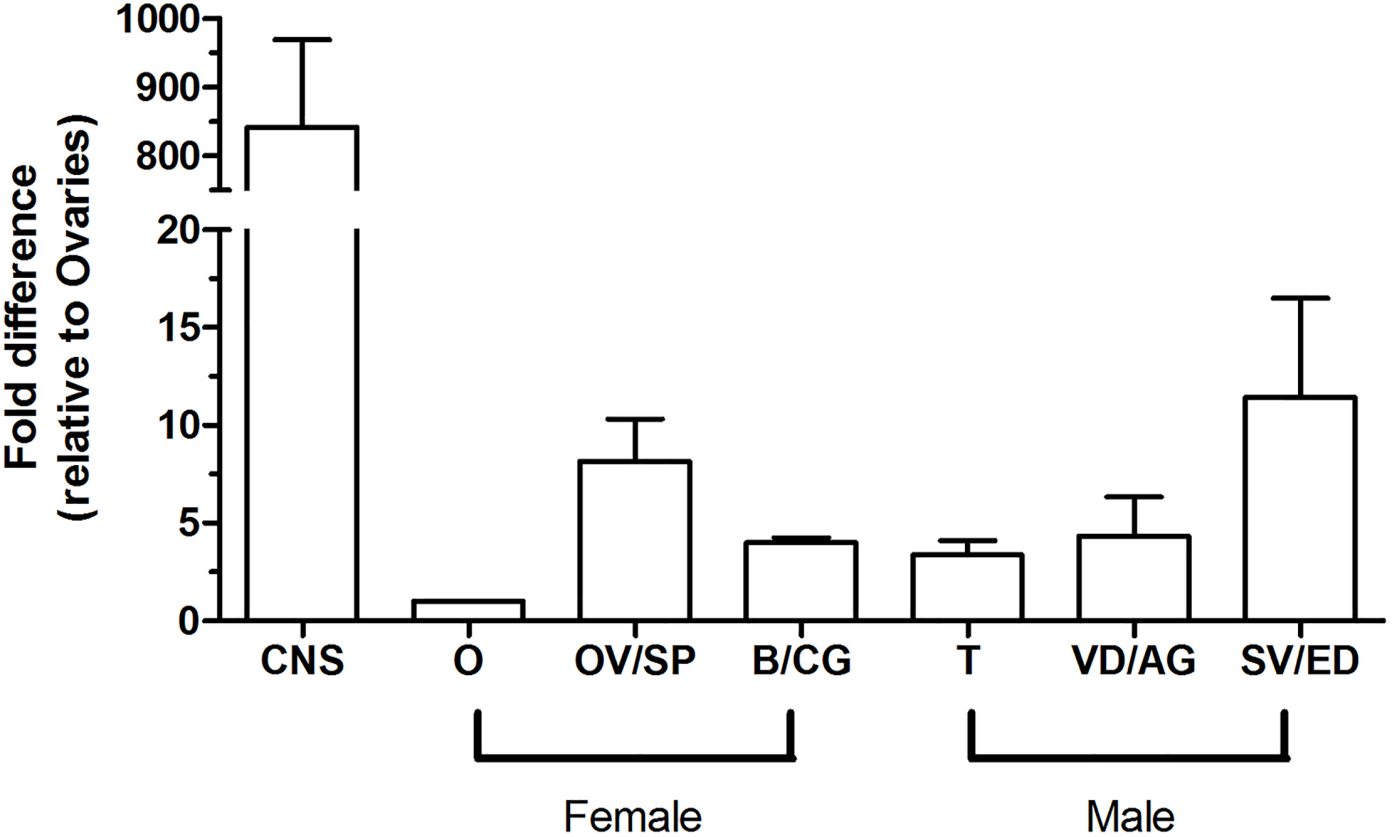
Spatial expression profile of the RhoprNPFR gene in the adult reproductive tract of *R*. *prolixus*. RhoprNPFR transcript levels were observed in adult male and female reproductive tissues and is shown relative to abundance in the female ovaries. Data points are mean ± standard error of the mean (SEM) of 3 biological replicates. Abbreviations: CNS, central nervous system; O, ovaries; OV/SP, oviducts and spermathecae; B/CG, bursa and cement gland; T, testes; VD/AG, vas deferens and accessory glands; SV/ED, seminal vesicle and ejaculatory duct.

Analysis of the fully developed adult reproductive system of males and females shows that the RhoprNPFR transcript is present throughout the whole reproductive system (Fig 6). Although transcript abundance in the adult CNS is approximately 850-fold greater relative to the levels found in the ovaries, RhoprNPFR transcript is present in the oviduct and spermathecae of females (~7.5-fold greater than levels in ovaries) as well as in the seminal vesicle and ejaculatory duct of males (~10-fold greater than levels in ovaries). Trace levels of mRNA were also found in the bursa and cement gland, testes, as well as the vas deferens and accessory glands (combination of transparent and opaque) in males.

### Distribution of RhoprNPFR mRNA in the CNS

**Fig 7:**
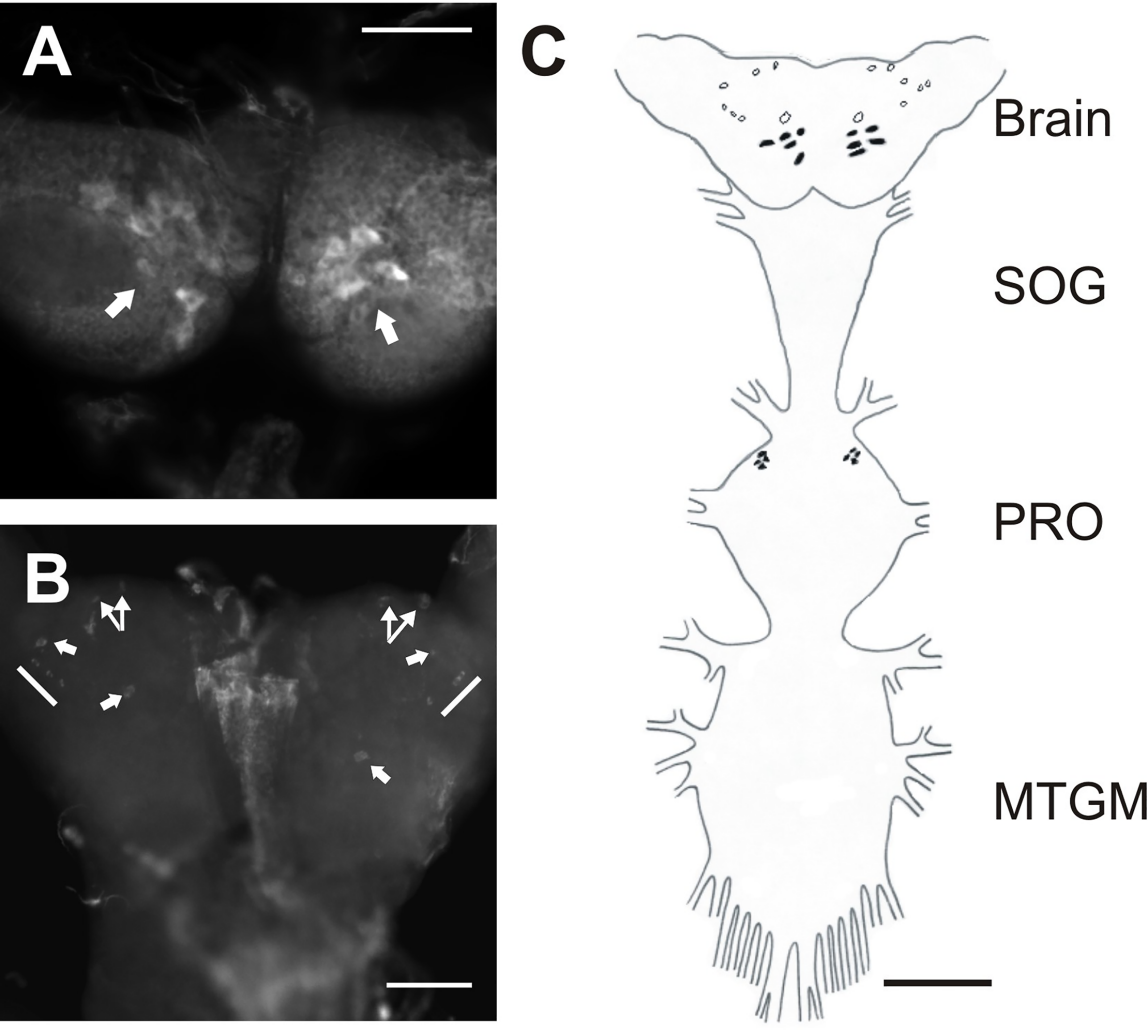
Cell-specific expression of the RhoprNPFR transcript in the CNS of 5^th^ instar *R*. *prolixus*. (A) A stacked image showing the 5 stained dorsal pairs (indicated by arrows), (B) and 7 ventral bilaterally-paired neurons in the brain (indicated by arrows). Scale bars for confocal images represent 100 μm. (C) A schematic map of the CNS portraying the distribution of all detected neurons that express the RhoprNPFR mRNA transcript, where dorsally located neurons are represented by closed circles and ventral neurons are open circles. The anterior of the prothoracic ganglion (PRO) contained 4 dorsolaterally paired cells that express RhorpNPFR. Scale bar for map represents 200 μm. Abbreviations: SOG, suboesophegeal ganglion; PRO, prothoracic ganglion; MTGM, mesothoracic ganglionic mass.

**Fig 8:**
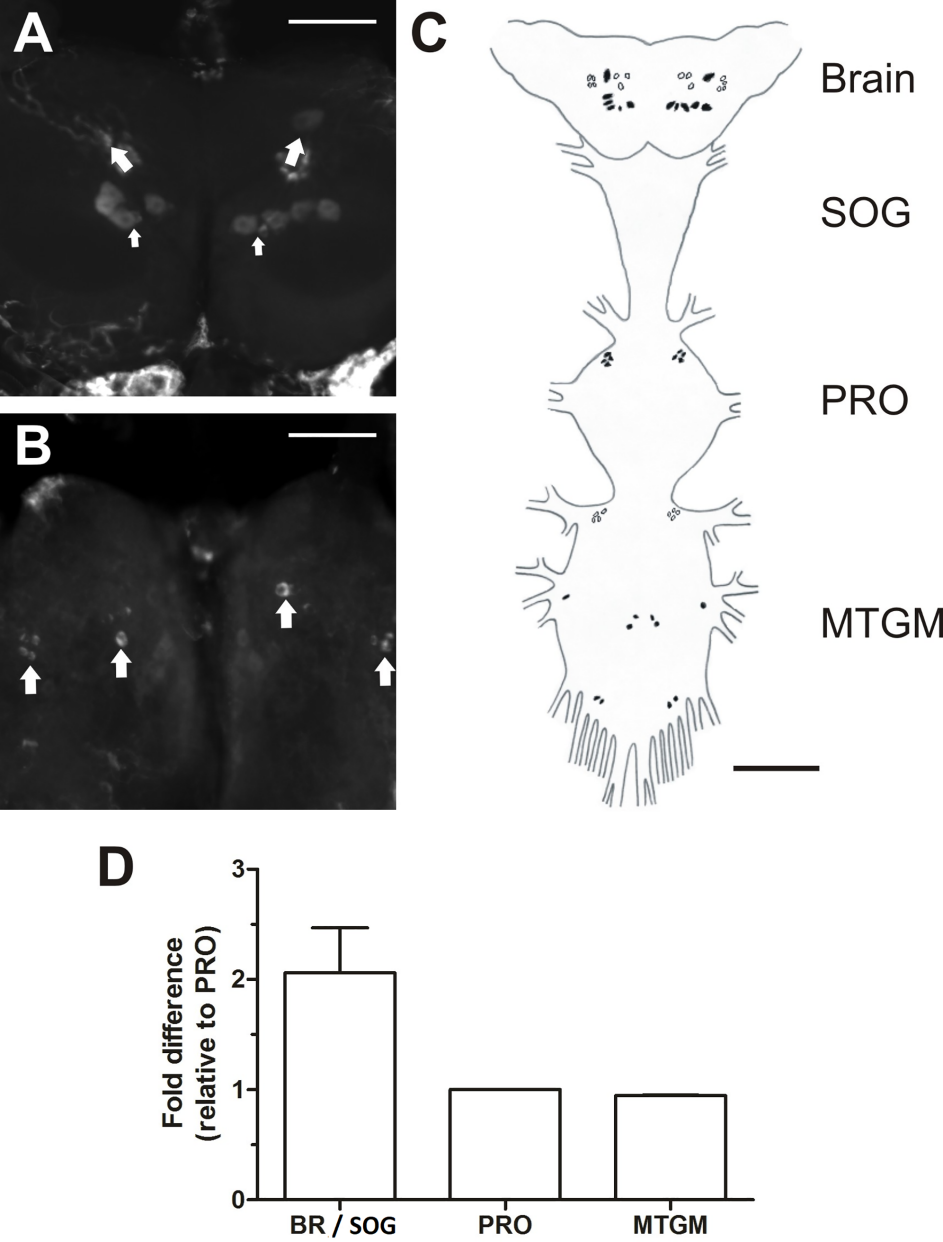
Cell-specific expression of the RhoprNPFR transcript in the CNS of adult *R*. *prolixus*. (A) A stacked image showing the 6 stained dorsal bilaterally-paired neurons in the brain (indicated by larger arrows), where one paired neuron (indicated by the smaller arrows) is substantially smaller in size than the remaining 5 neuron pairs. (B) Ventral view of the brain showing clusters of 3 bilaterally-paired medial neurons and 4 bilaterally-paired lateral neurons. Each cluster is indicated by a large arrow. Scale bars represent 100 μm. (C) A schematic map of the CNS outlining the distribution of all detected neurons that exhibit RhoprNPFR transcript expression, where dorsally located neurons are represented by closed circles and ventrally located neurons by open circles. Clusters of paired cells expressing RhoprNPFR transcript are present in the PRO as well as the MTGM. Scale bar for schematic map represents 200 μm. (D) Twice as much RhoprNPFR transcript is detected in the brain and SOG compared to the PRO and MTGM. Abbreviations: BR, brain; SOG, suboesophegeal ganglion; PRO, prothoracic ganglion; MTGM, mesothoracic ganglionic mass.

The NPF receptor transcript was shown to be highly expressed in the CNS of fifth instar and adult *R*. *prolixus* using qPCR. Fluorescent *in situ* hybridization was used to determine which neurons in the CNS express the transcript (Figs 7 and 8). RhoprNPFR is present in a group of five large bilaterally-paired dorsal medial neurosecretory cells (MNSCs) in the brain, approximately 30μm in diameter (Fig 7A). A large bilateral pair of cells is also present on the ventral surface of the brain, as well as a group of six smaller bilaterally-paired neurons that are localized more laterally, and are approximately 12.5μm in diameter (Fig 7B). Adults express the NPF receptor transcript in a group of six large bilaterally-paired dorsal MNSCs (Fig 8A). There were also two bilaterally-paired clusters of 3–4 cells located medially on the ventral surface of the adult brain.

Detection of the RhoprNPFR transcript was also present in the prothoracic ganglion within 4 dorsolateral paired neurons in fifth instars (Fig 7C) as well as adults (Fig 8C). Bilaterally-paired clusters of cells were also present in the adult mesothoracic ganglionic mass (MTGM) (Fig 8C). Examination of the relative transcript abundance in different regions of the adult CNS indicated that there is a two-fold higher abundance of RhoprNPFR transcript in the brain / suboesophegeal ganglion relative to the PRO and MTGM (Fig 8D).

### Presence of RhoprNPFR in the peripheral tissue of *R*. *prolixus*

**Fig 9:**
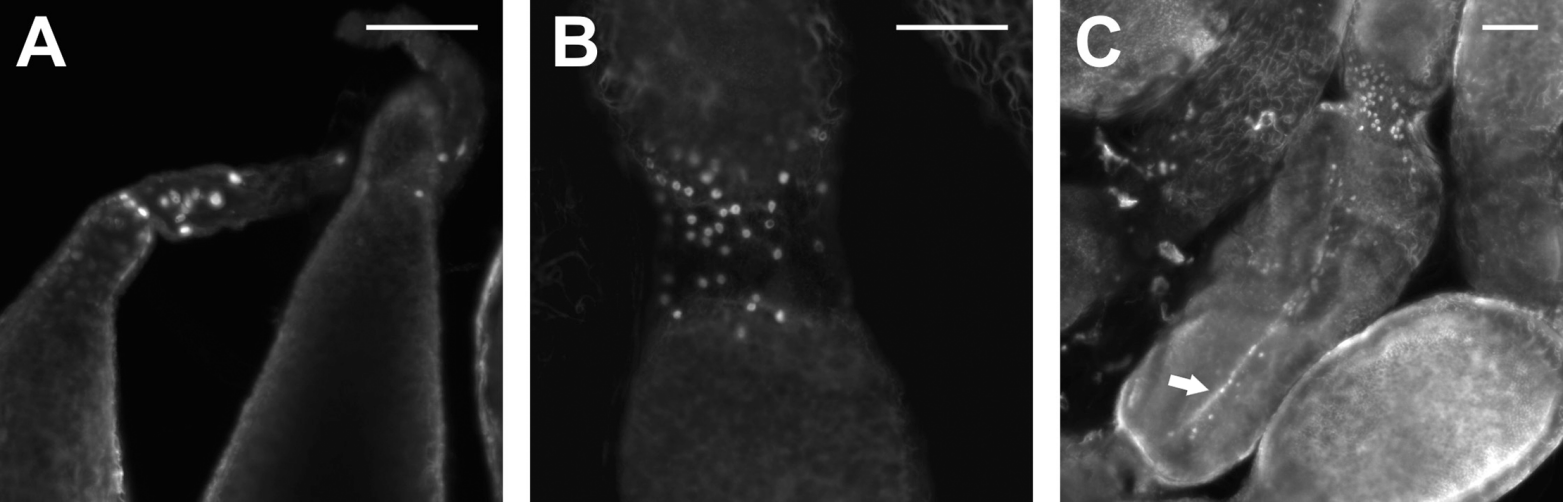
Stacked confocal images of accessory cells in the adult female ovarioles expressing RhoprNPFR using FISH. (A) A cluster of stained cells near the terminal filament and within the germarium, (B) in pre-follicular cells between developing oocytes and (C) along the nutritive cord of an ovariole expressing RhoprNPFR transcript (indicated by arrow). The nucleus of each cell exhibits no transcript staining. Scale bars represent 100 μm.

**Fig 10:**
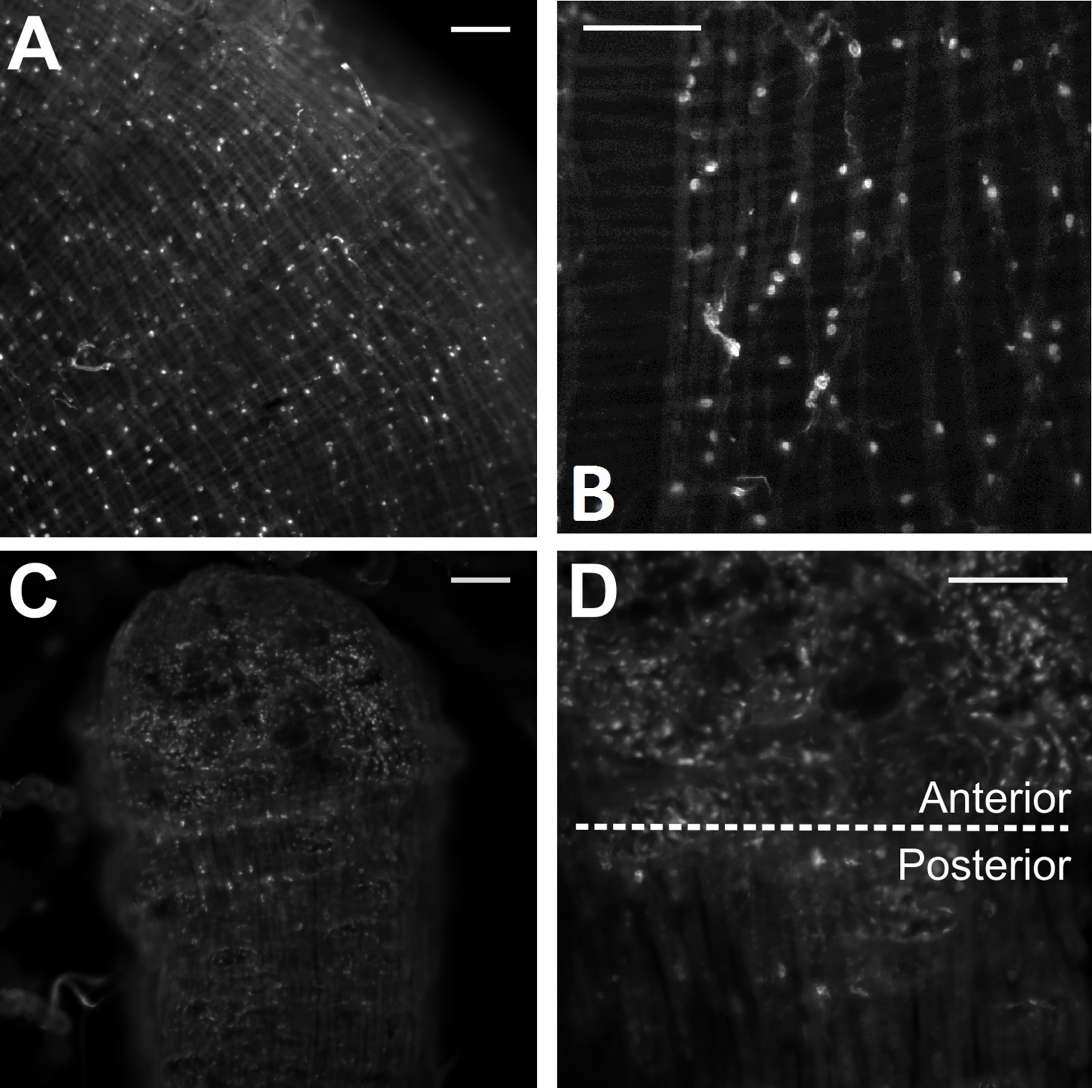
Stacked confocal images of stained cells along the fifth instar digestive tract of *R*. *prolixus* using FISH. (A) A stacked image showing stained endocrine cells along the anterior midgut of fifth instars. (B) A 20x magnified image shows that these putative endocrine-like cells present in the anterior midgut. (C) A 10x and (D) 20x stacked image showing differential staining along the hindgut of fifth instars where stained cells are more abundant on the anterior end than the posterior end of the hindgut. Scale bars represent 100 μm.

RhoprNPF receptor mRNA was present in putative pre-follicular cells within the germarium (Fig 9A) and between developing eggs of the adult ovariole (Fig 9B) as well as along the nutritive cord (Fig 9C). The transcript was absent from the adult digestive tract, but was found in putative endocrine-like cells of the fifth instar anterior midgut (Fig 10A and 10B). Some of these cells exhibit clear projections containing RhoprNPFR transcript (Fig 10B). Moreover, staining was present in small cells of the fifth instar hindgut with the greatest density of cells found in the anterior region of the hindgut (Fig 10C and 10D).

## Discussion

Only present in eukaryotes, G-protein coupled receptors have been associated with several diseases and have been a critical target for over 40% of the pharmacological medicinal drugs to date (see [26]). GPCRs also play a role in a plethora of physiological processes since they bind a broad range of ligands, including but not limited to, odour molecules, hormones, light-sensitive compounds, and neuropeptides (see [27]).

NPFR has been classified as a rhodopsin-like GPCR (see [2]). Although NPFR has been predicted *in silico* within many genomes, the NPF receptor gene has only been cloned in few insect species to date; the fruit fly, *D*. *melanogaster* [6], the African malaria mosquito, *A*. *gambiae* [7], the dengue and yellow fever vector mosquito, *A*. *aegypti* [8] and the locust, *L*. *migratoria* [9]. *RhoprNPFR* has an ORF that spans three exons where the fourth and sixth hydrophobic transmembrane domains span two different exons (exon 1–2 and 2–3, respectively). The ClustalW alignment of RhoprNPFR with related receptors shows that the *N*-linked glycosylation sites are predominantly conserved across species and are identified by an NxS/T domain [28]. After the translation of the nascent GPCR at the endoplasmic reticulum, covalent attachment of carbohydrate molecules at these specific motifs will aid in the overall protein folding and the formation of the correct tertiary biologically active conformation [28]. All seven hydrophobic transmembrane domains are also well conserved across arthropod GPCRs and are modeled to form α-helical secondary structures that arrange in a final barrel conformation (see [29]). Two cysteine residues that are completely conserved in arthropods are present within the first two extracellular loops (before the 3^rd^ and 5^th^ transmembrane domains) and form a disulfide bridge that is another classic identifier of GPCRs, but more critically, is important for the stabilization of the receptor’s structure (see [1]). On the cytosolic end of the third transmembrane domain there is a highly conserved DRY motif that is important for signaling (protein-protein interaction with G protein subunits) and intracellular trafficking (receptor internalization) [25]. This motif was similarly characterized in other GPCRs such as the crustacean cardioactive peptide receptor (CCAPR) in *R*. *prolixus* [30]. The importance of the DRY motif was defined in [25] by mutating the asparagine (D) and arginine (R) residues of a dopamine receptor and found that ligand-receptor interactions were abolished and that receptor internalization was effected differently based on the mutation [25]. The presence of (R/K)x(S/T) motifs in the cytosolic *C*-terminal chain allude to the use of either protein kinase C (PKC) or G-protein coupled receptor kinase (GRK) for phosphorylation when receptor internalization is required [31]. Phosphorylation of these residues results in the recruitment of β-arrestin which binds to other transport components such as clathrin and leads to endocytosis [31].

We demonstrated that the NPF receptor in *R*. *prolixus* belongs to a distinct monophyletic group of receptors, which is a sister group to NPF receptors identified in other insects, including dipterans [6, 7, 8], orthopterans [9] and coleopterans [32]. Interestingly, receptor sequences from another hemipteran insect, the brown plant hopper *N*. *lugens*, along with the more closely-related psocodean species (relative to dipterans), *P*. *humanus corporis*, reveal that they are members of the same distinct clade of NPF receptors, which agrees with the closer evolutionary relationships amongst these species [33]. One notable observation, however, is that the termite, *Z*. *nevadensis*, contains two NPF receptors that cluster into the two distinct monophyletic clades, suggesting two NPF receptor subtypes within this single species.

Spatial expression of the NPFR transcript has been observed in the African malaria mosquito across multiple developmental stages where greater expression of the receptor was found in adult females compared to males, implying a possible role in egg production or ovulation [7]. The RhoprNPFR transcript was localized to the head and abdomen, which was similar to that observed for the Ang-NPF transcript [7]. RhoprNPFR transcript abundance in fifth instar and adult stages was similar with a greater amount observed in the CNS and digestive tract, and expression in the reproductive tract. Cell-specific expression of RhoprNPFR mRNA was seen within cells along the anterior midgut of fifth instars. The CNS exhibited differential transcript expression of the receptor with higher transcript abundance in adults compared to fifth instar stage insects. Since RNA was extracted from a mixture of male and female insects, the differential expression between the sexes could not be inferred. However, expression of the NPF receptor mRNA within the oviduct/spermathecae of the adult female and seminal vesicle/ejaculatory ducts of the adult male strengthens the hypothesis of NPF being a potential regulator of reproductive processes. Although expression of RhoprNPFR transcript was found to be higher in fifth instar female ovaries in comparison to that of adults, it is important to note that the adult females used were unmated. Further studies will need to be carried out comparing unmated females and mated females in order to validate the importance of NPFR in *R*. *prolixus* reproduction.

Only one paper previously reported the cell-specific expression of NPFR in the brain of invertebrates, and the current study is the second to do so. *In situ* hybridization was used in [6] to localize DrmNPFR1within neurons of third instar larval *D*. *melanogaster* CNS, where numerous cells were detected in the brain as well as the ventral nerve cord. Putative endocrine-like cells in the anterior midgut of fifth instars were found to express the RhoprNPFR transcript indicating a potential regulatory role in this organ. RhoprNPFR transcript was localized in dorsal MNSCs in the brain of fifth instars and adults and was also observed in cells of the 4 week old unfed fifth instar hindgut, which were differentially distributed along the anterior and posterior regions of the hindgut. There is a greater density of labelled cells in the anterior portion of the hindgut (analogous to the ileum of other insects) and lower density on the posterior portion of the hindgut (analogous to the rectum). Previous studies have shown that DrmNPF inhibits *R*. *prolixus* hindgut contractions in fifth instars [34]. Further experiments comparing these results to fed fifth instars could clarify the importance of NPF in muscle relaxation. Although a greater expression of RhoprNPFR was found throughout the adult alimentary tract compared to that of fifth instars, FISH results showed no clear expression within cell bodies. This may reflect the greater sensitivity of qPCR versus that of FISH.

In a previous study, we proposed that RhoprNPF is capable of controlling certain aspects of reproduction since injection of the biologically-active truncated RhoprNPF resulted in a depletion of eggs present in the ovaries and an increase in the total number of eggs laid in *R*. *prolixus* [4]. This suggests that RhoprNPF may be involved in facilitating ovulation. We also found the presence of the RhoprNPF transcript within cells of the lateral oviducts [4]. Furthermore, other studies have shown that trNPF was responsible for oocyte maturation and development in female locusts [35, 36]. To examine the possible importance of NPF in female reproduction, we localized the RhoprNPFR transcript in the female reproductive tract. Putative pre-follicular cells within the germarium express RhoprNPFR mRNA, as do cells located between developing oocytes. In addition, RhoprNPFR transcript was also detected in the nutritive cord. The presence of RhoprNPFR transcript around developing oocytes suggests a role in egg production, regardless of the whether the female has been mated or not. Further work is needed to discover what role the NPF signaling pathway plays in the development of the oocyte or the embryo in unmated and mated females.

In conclusion, we cloned the cDNA of the RhoprNPF receptor and classified it as a GPCR. RhoprNPFR contained many of the defining characteristics of rhodopsin-type GPCRs, including N-terminal glycosylation sites, seven hydrophobic transmembrane domains, two extracellular cysteine residues involved in forming a structurally important disulfide bond, and the highly conserved DRY motif following the third transmembrane domain. RhoprNPFR is highly conserved among insect species, and most likely the receptor co-evolved with its ligand NPF across invertebrates, which is a commonly observed phenomenon [37]. The RhoprNPF receptor is expressed in the CNS and gut of *R*. *prolixus*, and there is a 10-fold increase in the expression of the receptor in the nervous system from fifth instars to adults. The mRNA transcript of RhoprNPFR was localized in five and six bilaterally-paired MNSCs in the fifth-instar and adult brain, respectively. RhoprNPFR expression was also localized in putative pre-follicular cells within the germarium of the developed telotrophic ovarioles of *R*. *prolixus*, and mRNA from nurse cells most likely is supplied to the growing oocytes via nutritive cords. Therefore, the RhoprNPFR may also play a role in oogenesis in *R*. *prolixus*, along with its role within the CNS and digestive system.

## Supporting information

S1 Fig Phylogenetic analysis of RhoprNPFR with related receptor sequences from other insects, including members of the NPF and sNPF receptor families. A rooted phylogenetic tree depicting the receptor sequence relationship inferred using the maximum likelihood method. The tree with the highest log likelihood (-7560.4321) is shown.The numbers at each node represent the percentage of replicate trees in which the associated receptor sequences clustered together in the bootstrap test (1000 replicates). The tree is drawn to scale, with branch lengths measured in the number of substitutions per site. The analysis involved 27 amino acid sequences. All positions containing gaps and missing data were eliminated. There were a total of 223 positions in the final dataset. Each receptor sequence is represented by the GenBank accession numbers and species name from which the sequence originated.
(PDF)
