# Supplementary figures and images for: Characterization and expression of a long neuropeptide F (NPF) receptor in the Chagas disease vector Rhodnius prolixus
Source: PLoS One. 2018 Aug 16;13(8):e0202425. doi: 10.1371/journal.pone.0202425 (PMC6095579; doi:10.1371/journal.pone.0202425)

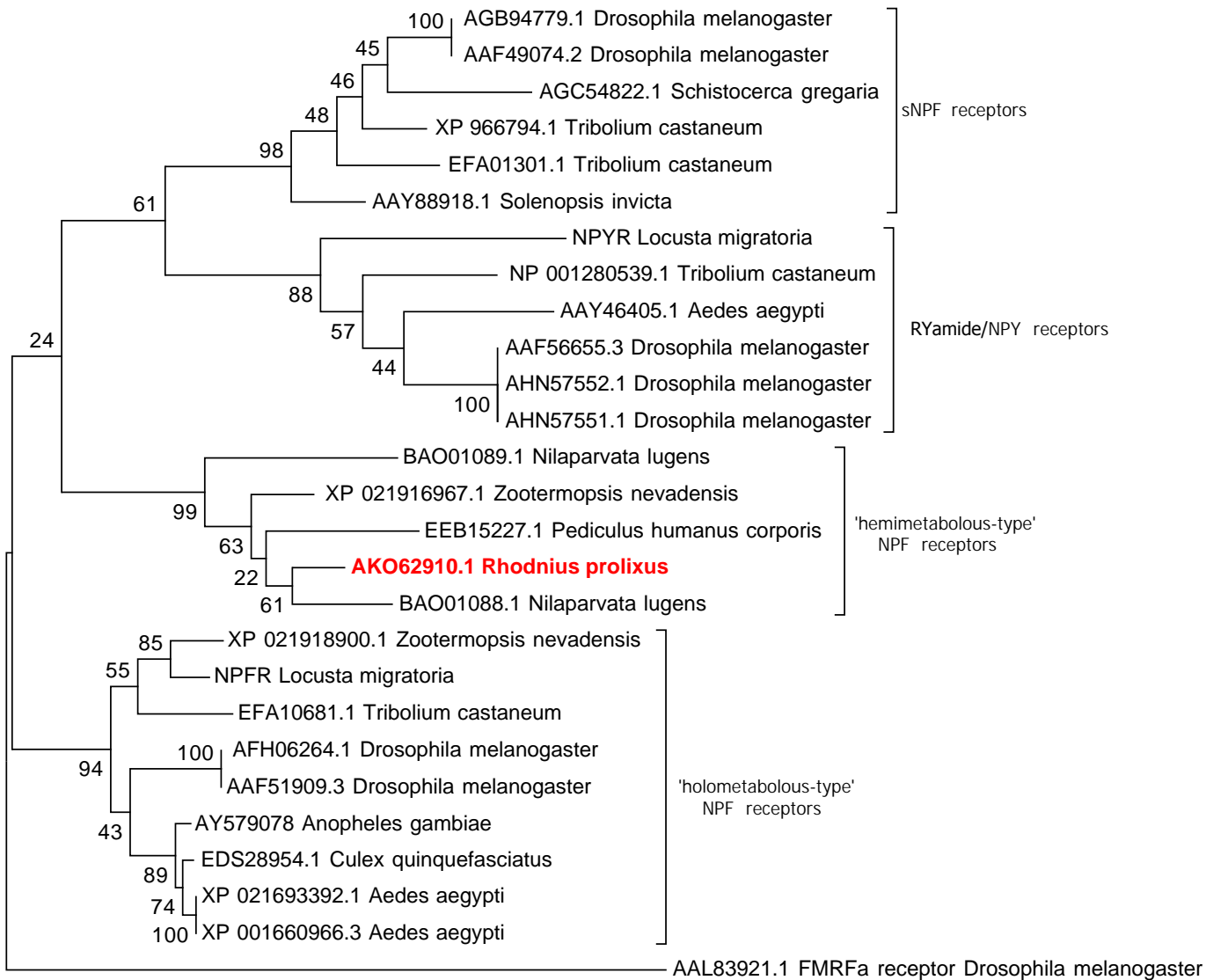

Supplement: S1 Fig — A rooted phylogenetic tree depicting the receptor sequence relationship inferred using the maximum likelihood method. The tree with the highest log likelihood (-7560.4321) is shown.The numbers at each node represent the percentage of replicate trees in which the associated receptor sequences clustered together in the bootstrap test (1000 replicates). The tree is drawn to scale, with branch lengths measured in the number of substitutions per site. The analysis involved 27 amino acid sequences. All positions containing gaps and missing data were eliminated. There were a total of 223 positions in the final dataset. Each receptor sequence is represented by the GenBank accession numbers and species name from which the sequence originated. (PDF) [file pone.0202425.s001.pdf]
